# Five-year overall survival following chemoradiation therapy for locally advanced cervical carcinoma in women living with and without HIV infection in Botswana

**DOI:** 10.1186/s13027-021-00389-w

**Published:** 2021-08-03

**Authors:** Emily MacDuffie, Memory Bvochora-Nsingo, Sebathu Chiyapo, Dawn Balang, Allison Chambers, Jessica M. George, Shawna Tuli, Lilie L. Lin, Nicola M. Zetola, Doreen Ramogola-Masire, Surbhi Grover

**Affiliations:** 1 Department of Radiation Oncology, Perelman School of Medicine, University of Pennsylvania, Philadelphia, USA; 2 Department of Oncology, Gaborone Private Hospital, Gaborone, Botswana; 3 Princess Marina Hospital, Gaborone, Botswana; 4 Department of Family and Community Health, Penn State Health St. Joseph, Hershey, USA; 5 Donald Bren School of Information & Computer Sciences, University of California Irvine, Irvine, USA; 6 Department of Radiation Oncology, University of Texas MD Anderson Cancer Center, Houston, USA; 7 Botswana-University of Pennsylvania Partnership, Gaborone, Botswana; 8 School of Medicine, University of Botswana, Gaborone, Botswana; 9 Department of Obstetrics and Gynecology, University of Botswana, Gaborone, Botswana

**Keywords:** Cervical cancer, Chemoradiation, HIV, Botswana, Sub-Saharan Africa, Low resource setting, Survival outcomes

## Abstract

**Purpose:**

To compare updated prospective 5-year survival outcomes of cervical cancer patients living with and without human immunodeficiency virus (HIV) infection who initiated curative chemoradiation therapy (CRT) in a resource-limited setting.

**Methods & Materials:**

Women in Botswana with locally advanced cervical cancer were enrolled in a prospective, observational, cohort study from July 2013 through January 2015. Survival outcomes were analyzed after 5 years of follow-up.

**Results:**

This cohort included 143 women initiating curative CRT. Sixty-seven percent (*n* = 96) of cohort were women living with HIV (WLWH), all of whom were receiving antiretroviral therapy (ART) at the time of treatment initiation and boasted a median CD4 count of 481 cells/μL (IQR, 351-579 μL). The 5-year overall survival (OS) rates were 56.8% (95% CI, 40.0–70.5%) for patients without HIV infection and 55.1% (95% CI, 44.2–64.7%) for WLWH (*p* = 0.732). Factors associated with superior 5-year OS on multivariate analyses included baseline hemoglobin > 10 g/dL (hazard ratio (HR) 0.90, 95% CI, 0.83–0.98, *p* = 0.015), lower stage at diagnosis (stage I and II vs. III and IV) (HR 1.39, 95% CI 1.09–1.76, *p* = 0.007), and higher EQD2 (HR 0.98, 95% CI 0.97–0.99, *p* = 0.001).

**Conclusions:**

Five-year OS was not impacted by HIV status in this population of WLWH with well-managed infection who initiated curative treatment for cervical cancer in Botswana. Regardless of HIV status, hemoglobin levels and stage at diagnosis were associated with survival. These findings suggest that treatment for cervical cancer in WLWH with well-controlled infection need not be altered solely due to HIV status.

## Introduction

Globally, cervical cancer is the fourth most common cancer in women in terms of both incidence and mortality, with 85% of disease burden falling on women in low- and middle-income countries [1]. Chronic persistent human papillomavirus (HPV) infection is known to be the most critical risk factor for the development of cervical cancer [2], and co-infection with human immunodeficiency virus (HIV) increases the likelihood of cervical cancer development [3].

In Botswana, a middle-income country in sub-Saharan Africa (SSA), cervical cancer is the leading cancer type in females in terms of both incidence and mortality [1]. Additionally, HIV is hyperendemic in the nation with peak prevalence of 50.6% in females 35–39 years old [4]. Since initiating a publicly-funded antiretroviral therapy (ART) program in 2002, an aging population living with HIV infection has emerged in Botswana, and, subsequently, the incidence of cervical cancer has increased by 3.0% annually [5].

For individuals who have been diagnosed with locally advanced cervical cancer, the global standard-of-care includes concurrent radiotherapy and cisplatin-based chemotherapy [6]. The potential effects of HIV infection on cervical cancer survival and treatment toxicity, however, were previously poorly described. Thus, we have sought to elucidate these potential effects by prospectively comparing a cohort of WLWH receiving chemoradiation (CRT) to those without HIV infection receiving the same treatment in Botswana.

Our previous report comparing survival rates after two years of follow-up found that HIV status was not associated with 2-year overall survival (OS) in WLWH on ART who initiated curative CRT [7]. This letter serves to expand upon our previous results and compares survival in WLWH and without HIV infection after concluding 5 years of follow-up.

## Methods & materials

This observational cohort study prospectively enrolled women with locally advanced cervical cancer who were initiating curative chemoradiation at the only radiation facility in Gaborone, Botswana between July 2013 and January 2015. Treatment with curative intent was defined as a prescription of 45 to 50 Gray (Gy) of whole-pelvis RT, weekly concurrent cisplatin (35–40 mg/m2) for 5 cycles, and high-dose-rate brachytherapy (7 Gy in 3–4 fractions or 6 Gy in 4–5 fractions). Five-year follow-up data collection concluded in February 2020. For complete method details see Grover et al. [7]

Upon initial enrollment, patient data including demographics and clinical characteristics were collected via patient interview and chart abstraction. Chemotherapy cycles were recorded weekly. Total radiation dose received at the conclusion of treatment was calculated via the radiobiological equivalent dose (EQD2) formula [8].

Follow up was conducted by phone every three months. If patients were not available, next of kin were contacted and medical records were reviewed for documentation indicating last appointment or death.

This study was reviewed and approved by an institutional review board in the USA and the Ministry of Health in Botswana.

## Results

For complete demographics, clinical characteristics, and treatment parameters, please refer to Grover et al. [7] Briefly, a majority of patients were WLWH (*n* = 96, 67%) with median CD4 count of 481 cells/μL (interquartile range (IQR), 351-579 μL) and median time on ART of 84 months (IQR, 24–120 months). The median age of WLWH was 43.4 years, significantly younger than those without HIV infection at 53.9 years (*p* = 0.004). Patients who presented with stage I, II, III or IV disease accounted for 14.9, 60.6, 24.5, and 0% of the WLWH and 6.4, 51.1, 38.3, and 4.3% of the cohort without HIV (*p* = 0.04). Baseline hemoglobin significantly differed between WLWH and without HIV infection (*p* = 0.04), with 10.6 g/dL (95% confidence interval (CI), 9.2–12.1) and 11.3 g/dL (95% CI, 10.5–13), respectively. Treatment received by patients did not differ by HIV status, with WLWH receiving a median dose of 79.8 Gy (IQR, 74–79.8 Gy) and 58.3% (*n* = 56) receiving ≥4 cycles of chemotherapy and patients without HIV infection receiving 79.8 Gy (IQR, 68.8–79.8 Gy) and 53.2% receiving ≥4 cycles. OS at 2 years was 65.5% (95% confidence interval [CI] 56–73%) overall, 65.0% (95% CI 54–74%) for WLWH, and 66.0% (95% CI 49–79%) for patients without HIV infection.

**Fig. 1:**
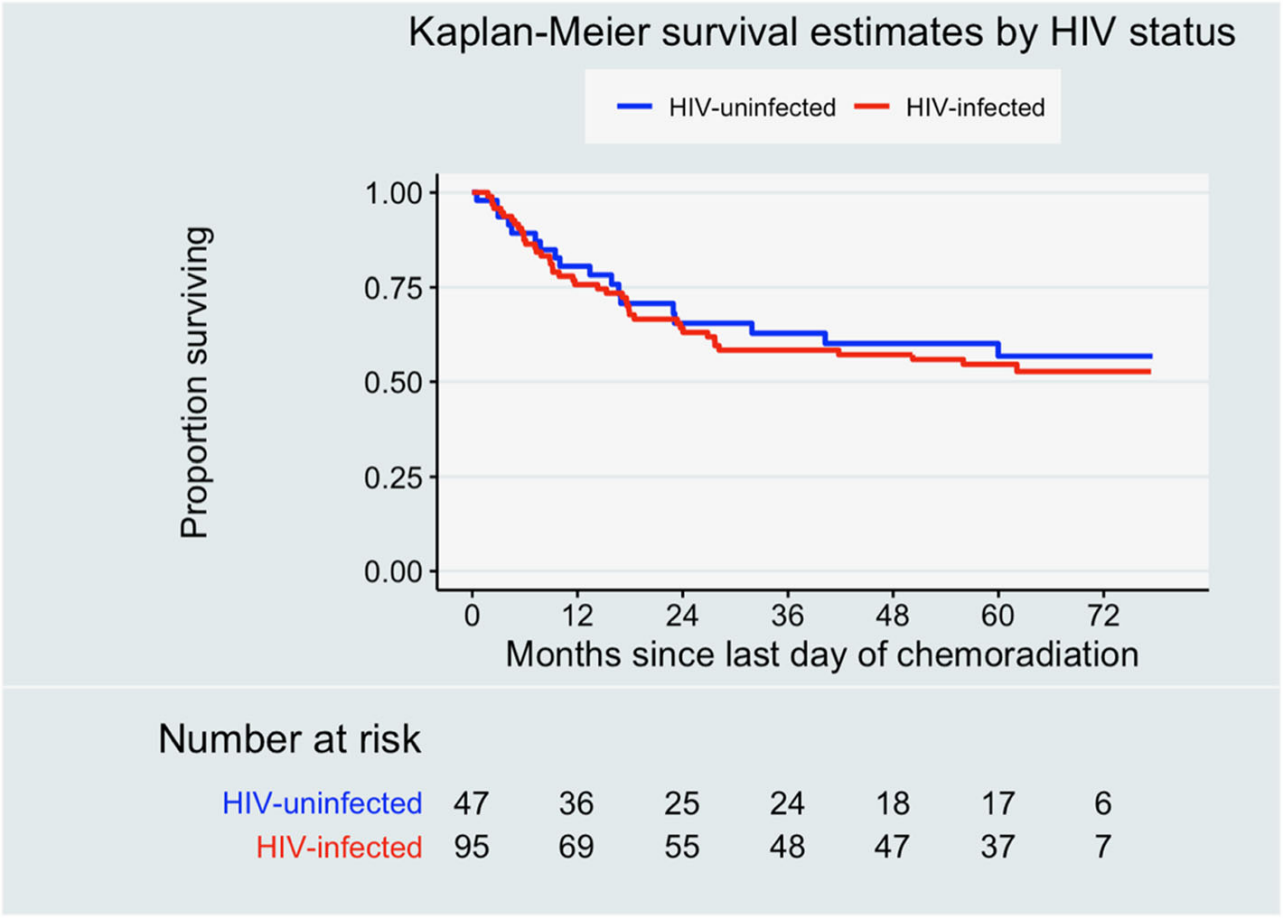
Overall survival by human immunodeficiency virus (HIV) status for cervical cancer patients treated with curative-intent chemoradiation

Analysis at 5 years had a median follow-up time for all patients of 37.6 months (63.0 months for living patients). Five-year OS was 55.6% (95% CI, 46.6–63.7%) for all patients, 56.8% (95% CI, 40.0–70.5%) for patients without HIV infection, and 55.1% (95% CI, 44.2–64.7%) for WLWH (*p* = 0.732) (Fig. 1). On univariate analysis, baseline hemoglobin level > 10 g/dL was associated with better OS, while poorer OS was associated with higher initial stage at diagnosis (stage III and IV) and receipt of < 2 chemotherapy cycles (Table 1). After adjusting for HIV status, age, stage, number of chemotherapy cycles received, baseline hemoglobin, and total radiation dose (EQD2), multivariate analysis demonstrated that baseline hemoglobin ≥10 g/dL remained associated with improved survival and advanced stage (stage III and IV) remained associated with poorer survival (Table 1). Notably, neither univariate nor multivariate analyses found that HIV status was associated with poorer survival in this cohort.

**Table 1:** Predictors of death at five years for cervical cancer patients in Botswana who initiated curative chemoradiation treatment

Variable	Univariate		Multivariate	
	HR	95% CI	HR	95% CI
HIV				
Uninfected	Reference			
Infected	1.101311	.6339304 1.913278	1.250879	.657968 2.378075
Stage				
IA IB	Reference			
IIA IIB	3.229473	.991521 10.51868	2.72587	.802824 9.255287
IIIA IIIB IV	**4.204608**	**1.255063 14.08593**	**3.747349**	**1.08663 12.9231**
Age				
<40	Reference			
40-59	1.225026	.6760837 2.219677	1.617596	.8523011 3.070061
>60	1.445736	.6559022 3.186681	1.953187	.8017136 4.758482
Baseline Hb				
≤10	Reference			
>10	**0.4893405**	**.292142 .8196497**	**0.4624402**	**.256955 .8322505**
Chemo cycles				
1	**2.442394**	**1.191725 5.005592**	1.992836	.9455122 4.200256
2	1.039553	.4325541 2.498349	0.7922305	.3125914 2.007826
3	1.202074	.6371535 2.26787	1.269207	.659052 2.444246
≥4	Reference			
Final EQD2				
<75	Reference			
≥75	1.011468	.9875201 1.035996	1.01332	.9895877 1.037622

## Discussion

Understanding the impact of HIV infection on cervical cancer treatment outcomes is critical to providing optimal care to women around the globe. Our analysis of this prospective cohort with locally advanced cervical cancer in Botswana living with well-controlled HIV infection previously demonstrated that 2-year OS was not impacted by HIV status^7^. Improved 2-year survival was associated with baseline hemoglobin level > 10 g/dL, receipt of a total radiation dose ≥75 Gy, and age < 40 years. WLWH were not preferentially prescribed non-curative treatment compared to those without HIV in this cohort [9]. This follow-up analysis at 5 years continues to demonstrate that HIV status does not impact survival outcomes of women with cervical cancer treated in Botswana. While 34.5% of patients were deceased at two years, only an additional 9.9% were deceased at five years, as would be expected in cervical cancer where the majority of deaths occur in the first two years [10]. Similar to our previous findings, hemoglobin levels > 10 g/dL remained an important predictor of survival at 5 years.

Our updated analysis provides an important addition to the available literature regarding long-term cervical cancer outcomes for WLWH with well-managed infection [11]. Since the publication of our 2-year data in January 2018, only one study has published data with extensive clinical follow-up. Simonds and colleagues [12] conducted a study in South Africa between 2007 and 2011 and concluded that HIV infection was associated with poorer 5-year survival. However, the proportion of WLWH infection in the cohort was small (14%). Given that the period of data collection occurred earlier in the era of ART availability compared to our study, 40% of patients were not taking ART at the time of cancer diagnosis, and the median CD4 count was just over 350 cells/uL. These differences in patient populations are similar to those of other studies, including Coghill et al [13], Dryden-Peterson et al [14], and Ferreira et al [15] that have reported findings discordant with our own. Thus, the data presented here demonstrate the importance of medical management of HIV before cervical cancer is detected and treated as well as underscore the critical nature of public health efforts to increase HPV vaccination, broaden screening efforts, and provide sufficient ART to all WLWH. Furthermore, our 5-year results indicate that WLWH with well-managed infection have outcomes that do not differ from those without HIV infection and therefore should be prescribed curative standard-of-care treatment for locally advanced cervical cancer.

## Data Availability

The datasets used and/or analysed during the current study are available from the corresponding author on reasonable request.

## Abbreviations

HIV: human immunodeficiency virus
WLWH: women living with HIV
ART: antiretroviral therapy
HPV: human papillomavirus
SSA: sub-Saharan Africa
CRT: chemoradiation
OS: overall survival
Gy: Gray
EQD2: radiobiological equivalent dose
IQR: interquartile range
CI: confidence interval
